# Comparison of glucose tolerance in renal transplant recipients and hemodialysis patients

**DOI:** 10.1186/1471-2369-5-11

**Published:** 2004-09-08

**Authors:** Hassan Argani, Alireza Noorazarian, Mohammad Rahbaninobar, Mohammad Noori, Hamid T Khosroshahi

**Affiliations:** 1 Nephrology Division of Emam Hospital, Tabriz university of medical sciences, Tabriz, Iran; 2 Drug Applied Research Center, Tabriz university of medical sciences, Tabriz, Iran; 3 Biochemistry Laboratory, Emam Hospital, Tabriz university of medical sciences, Tabriz, Iran; 4 Biochemistry Division of Medical Faculty, Tabriz university of medical sciences, Tabriz, Iran

## Abstract

**Background:**

Impaired glucose tolerance is a risk factor for atherosclerosis in hemodialysis patients and renal transplant recipients.

**Methods:**

To check the relationship of impaired glucose tolerance with the other atherosclerotic risk factors, fasting blood sugar and the standard two hour glucose tolerance test, serum tryglyceride, serum cholesterol, cyclosporine through level (in renal tranpslant recipients) and hemoglobin A1C were measured in 55 stable renal transplant recipients, 55 hemodialysis patients and 55 healthy controls with similar demographic characteristics. Patients with diabetes mellitus and propranolol consumers were excluded. The mean age and female to male ratio were 39 +/- 7 years and 23/22, respectively.

**Results:**

Four of the renal transplant recipients and twelve of the hemodialysis patients had impaired glucose tolerance. Significant linear correlation was observed with body mass index and IGT only in hemodialysis patients (r = 0.4, p = 0.05). Glucose tolerance also had a significant correlation with triglyceride levels (217.2 +/- 55 mg/dl in hemodialysis patients vs. 214.3 +/- 13 mg/dl in renal transplant recipients and 100.2 +/- 18 mg/dl in control groups, p = 0.001). The glucose tolerance had significant relationship with higher serum cholesterol levels only in the renal transplant recipients (269.7 +/- 54 in renal transplant recipients vs. 199.2 +/- 36.6 mg/dl in hemodialysis and 190.5 +/- 34 mg/dl in control groups, p = 0.0001). In the renal transplant recipients, a linear correlation was observed with glucose tolerance and both the serum cyclosporine level (r = 0.9, p = 0.001) and the hemoglobin A1C concentration (6.2 +/- 0.9 g/dl). The later correlation was also observed in the hemodialysis patients (6.4 +/- 0.7 g/dl; r = 67, p = 0.001).

**Conclusions:**

We conclude that although fasting blood sugar is normal in non-diabetic renal transplant and hemodialysis patients, impaired glucose tolerance could be associated with the other atherosclerotic risk factors.

## Background

Mortality and morbidity due to cardiovascular diseases are frequent in patients with diabetes mellitus and high prevalence of diabetes and cardiovascular disease, also, are observed in patients with end-stage renal disease treated by renal replacement therapy, either renal transplantation (RT) and dialysis [1]. Although uremia is typically associated with impaired glucose metabolism via multiple mechanisms [2-4], hemodialysis improves, although not completely, the uremic induced glucose impairment [5-7]. Impaired glucose metabolism is also a common and an important problem after RT. By improvement of immunosuppression after RT, the incidence of post transplant diabetes (PTDM) has been decreased from 41% to 2.5% [8,9]. Although we routinely screen and treat only full-blown diabetes at the post transplant periods, an overlooked aspect is the impaired glucose tolerance, which may be a risk factor to induce atherosclerosis. Impaired glucose tolerance *de novo*, may be a risk factor of post-transplantation mortality and morbidity [10]. Although increased levels of glycosylated hemoglobin (HbA1C) and lipid concentrations have been shown in hemodialysis patients [11] and renal transplant recipients [12] with diabetes, their impairment is not clear in the both groups with impaired glucose tolerance without apparent diabetes mellitus. In this study we investigated glucose tolerance and lipid profiles in non-diabetic hemodialysis and renal transplant patients.

## Methods

We selected fifty five RT recipients with more than one year of good renal allograft function (serum Cr < 1.5 mg/dl), under conventional triple therapy composed of cyclosporine A (CsA), azathiopurine and prednisolone. Their allograft sources were living donors. Fifty five stable HD patients and another fifty five healthy controls (C), were also enrolled in this study. The mean age (39 ± 7 years), sex (F/M ratio was 33/22), body mass index (BMI) 24.7 ± 1.28 kg/m^2^) were similar in the three groups (see table 1). Patients with diabetes mellitus and propranolol consumers were excluded.

**Table 1:** Demographical, biochemical, hematological and therapeutical factors in hemodialysis patients and renal transplant recipients.

	HD	RT	Control
Age (years)	48 ± 3	46 ± 4	47 ± 4
Male/female ratio	33/22	33/22	33/22
BMI (Kg/m^2^)	24.6 ± 1.4	23.8 ± 1.2	23.6 ± 1.3
Cholesterol (mg/dl)	199.2 ± 36.6	269.7 ± 54	190.5 ± 34
Triglycerides (mg/dl)	217.2 ± 55	214.3 ± 13	100.2 ± 18
HgA1C (g/dl)	6.42 ± 0.7	6.2 ± 0.9	5.7 ± 0.7
Hb level	10.9 ± 0.8	12.4 ± 1.1	13.3 ± 0.8
Therapy with vitamin D3 0.5 μg/day (number)	25	--	--
Impaired glucose tolerance (number)	12*	4**	--

*Significant correlation with BMI, serum triglycerides and HgA1C

** Significant correlation with serum cholesterol, CysA concentration and HgA1C

The levels of serum triglyceride, cholesterol (measured by enzymatic spectrophotometry)[13], CsA (measured by ELISA in whole blood, only in renal transplant recipients) and glycosylated haemoglobin concentration (Hb A1c) (measured by column chromatography) were measured after 10 hours fasting (in the hemodialysis group, in the early morning before hemodialysis). Fasting blood sugar and the standard 2 hours glucose tolerance test (after ingestion of 75 g of glucose) were detected in the three groups by spectrophotometry. Statistical analysis was performed by Kuruskal wallis, U-Mann Whitney, multiple comparison and regression correlation coefficient tests, using SPSS 10.05.

## Results

**Figure 1:**
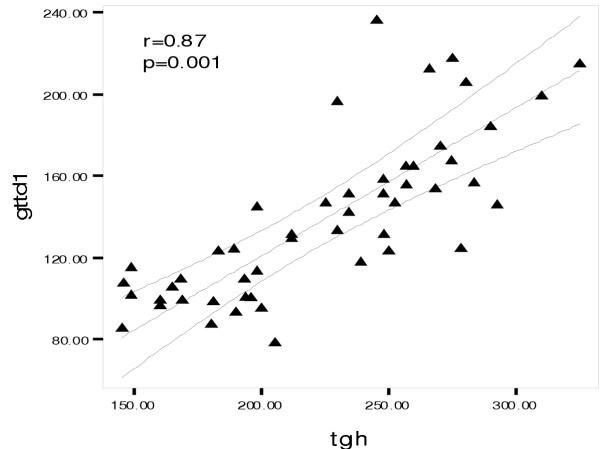
GTT has a linear relationship with BMI in hemodialysis patients. Impairment of GTT is more significant in the hemodialysis patients with higher BMI. Gtt2 = glucose tolerance test at the second hours of 75 gr oral glucose. bmih = body mass index in hemodialysis patients

**Figure 2:**
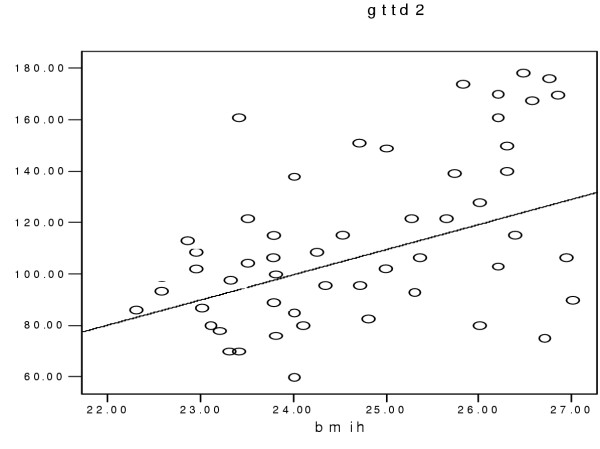
The glucose tolerance in the HD patients had a significant linear correlation with the level of serum triglycerides. gttd1= glucose tolerance test in dialysis patients, tgh= serum concentration of triglyceride in hemodialysis patients.

**Figure 3:**
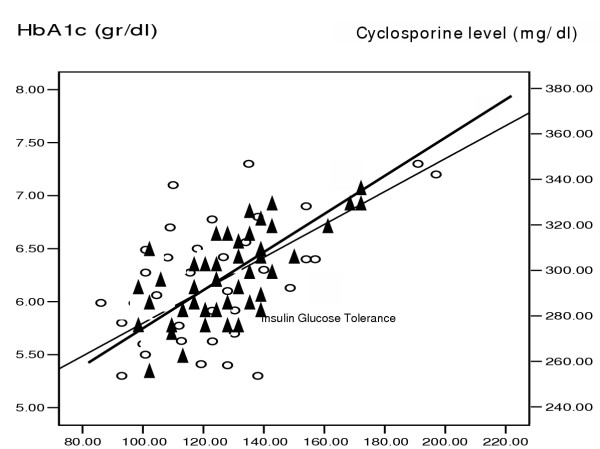
Cyclosporine level and HbA1c have correlations with the IGTT in RT recipients ▲ = Serum Cyclosporine level ○ = HbA1c concentration

On the basis of WHO classification [14], four of our (7.5%) renal transplant recipients and twelve (22%) of the hemodialysis patients had impaired glucose tolerance, i.e. the 2 hour of glucose tolerance test was between 140 and 200 mg/dl. It was more obvious at the end of the second hour of GTT. Although BMI was roughly similar in the three groups (Table 1), a significant linear correlation was observed between BMI and impaired glucose tolerance only in HD patients (r = 0.4, p = 0.05) (fig 1), but not in the RT recipients. The glucose tolerance (especially at the first hour) in the HD patients had a significant linear correlation with the level of serum triglycerides (r = 0.87, p = 0.001) (Fig 2). Serum triglyceride concentration was 217.2 ± 55 mg/dl in HD vs. 214.3 ± 13 mg/dl in RT and 100.2 ± 18 mg/dl in C groups, (p = 0.001). On the other hand the four RT recipients with IGTT (i.e. 100% of RT recipients with IGTT) had the higher serum cholesterol levels (308.4 ± 24.4 mg/dl)) compared with the remaining RT recipients with normal GTT (248.7 ± 55.6 mg/dl) with p = 0.031 (table 2). The mean of serum cholesterol was 269.7 ± 54 mg/dl in RT vs. 199.2 ± 36.6 mg/dl in HD and190.5 ± 34 mg/dl in C groups (p = 0.0001). A linear correlation was observed between impaired GTT and both of the serum Cyclosporine level (r = 0.9, p = 0.001) and HbA1c in RT recipients (Fig 3). The mean of HbA1c was 6.2 ± 0.6 gr/dl in the RT recipients with normal GTT vs. 4.34 ± 0.26 g/dl in the RT recipients with IGGT (p < 0.001, table 2). The later correlation was also observed in HD patients, in whom the mean of HbA1C level was 6.4 ± 0.7 gr/dl in the group (r = 67, p = 0.001). In contrast of a close relationship of IGTT and higher HbA1c, the gender, age, times after transplantation and BMI did not impact on IGTT in RT recipients. Although in logistic regression analysis higher serum level of cyclosporine was correlated with increased GTT impairment, we could not evaluate the implication of corticosteroids on this test, because all of the 55 RT recipients were received prednisolone at a doses of 5 to 10 mg/day.

**Table 2:** Impaired glucose tests in HD and RT recipients have higher values of serum triglyceride, serum cholesterol and cyclosporine concentration than patients with normal glucose tolerance tests.

	no. of cases	Serum Triglyceride(mg/dl)	Serum Cholesterol(mg/dl)	HbA1c (gr/l)	Cyclosporine (mg/dl)
RT recipients with IGTT	4	231.4 ± 150	308.4 ± 24.4	7.34 ± 0.26	320.4 ± 36.6
RT Recipients With normal GTT	51	201 ± 75	248.7 ± 55.6	6.2 ± 0.6	295.1 ± 29
		***P = 0.59***	***P = 0.02***	***P = 0.001***	***P = 0.2***
HD patients with IGTT	12	272.1 ± 41.3	201.1 ± 39	7 ± 1	
HD patients with normal GTT	43	195.9 ± 45	198.5 ± 36.8	5.9 ± 0.7	
		***P = 0.001***	***P = 0.87***	***P = 0.007***	
Controls	55	100.2 ± 18	190.5 ± 34	5.7 ± 0.7	

## Discussion

Impaired glucose tolerance occurs in about 50% of patients with chronic renal failure (CRF) patients. It is due to multiple factors, which the two most important of them being insulin resistance at target organs and impaired insulin secretion from the pancreas [15]. Insulin sensitivity would be reduced by up to 60% in non-diabetic patients with CRF before dialysis [16]. Marked improvement in insulin sensitivity and consequently glucose tolerance has been reported in non-diabetic patients after 10 weeks of HD, although they are not completely returned to normal [15]. Thereby, impaired glucose tolerance during HD is secondary to non-effective removable toxins by HD compared with peritoneal dialysis. In the latter more effective removal of middle molecule toxins causes better glucose tolerance, although glucose rich dialyzet solution is used [16]. The other causes of impaired glucose tolerance in HD patients may be secondary to metabolic disturbances, such as anemia [17], malnutrition [18] and vitamin D3 deficiency [19]. Although all of our HD patients had normochromic-normocytic anemia, the severity was not proportionate with impaired glucose tolerance (The data has not been shown). The patients were well nourished and were under treatment with daily oral vitamin D3 (Rocaltrol), 0.5 micrograms per day. So malnutrition and vitamin D3 deficiency could not to contribute to impaired glucose tolerance in our HD patients. Impaired glucose tolerance was also observed in 7.5% of our RT recipients. All of the presumed risk factors for post transplant diabetes mellitus such as old age [18], family history of any known diabetes mellitus in their first relatives[21], cadaveric allografts [22] and obesity did not exist in the patients. Previously Boudreaux et al. [23] reported that those patients who weighed more than 70 kg had a higher incidence of post transplant diabetes mellitus (PTDM). A relative risk of 1.4 for developing PTDM for every 10 kg increase in body weight more than 60 kg has been shown [12]. Although in our study obese patients (BMI > 30 kg/m2) were not included in the both groups, a correlation was observed between impaired glucose tolerance and higher BMI in our HD patients. In RT recipients, the major risk factor for impaired glucose tolerance was immunosuppressive therapy. Through using higher doses of CsA and corticosteroids, PTDM was previously more common, but the complication has been decreased to 2–5% in FK506-based immunosuppressive protocols [24,25]. Although this relatively uncommon complication is a major cause of post-transplant mortality and morbidity, even minor glucose intolerance is associated with an increased long-term risk for cardiovascular disease [26]. The importance of impaired glucose tolerance should not be underestimated in these patients with high risk of atherosclerosis. Hyperlipidemia, another risk factor for atherosclerosis, on one hand accompanies the impaired glucose tolerance observed in the HD and RT patients and on the other hand increases the risk of atherosclerosis induced by impaired glucose tolerance. As reported previously, a tendency to higher pre-transplantation serum triglyceride concentration was associated with post-transplantation impaired glucose tolerance [27].

Hypertriglyceridemia is common complication in dialysis patients. In non-transplant populations it is regarded (along with low HDL cholesterol levels) as a prominent feature of insulin resistance syndrome, and also is a cardiovascular risk factor in organ transplant recipients [28]. Our study confirmed the relationship between impaired glucose tolerance and triglyceride levels in HD patients, and between impaired glucose tolerance and cholesterol levels in RT recipients. The latter was also accompanied by a higher level of HgA1C. Commonly used tests of HgA1C may be unreliable in patients with end-stage renal disease because of the presence of anemia, shortened red blood cell survival, and assay interferences from uremia. But HgA1C in the range of 6% to 7%, as was found in our study, estimates glycemic control within the range of patients without severe renal impairment [1]. So in the range of mild to moderate increased HgA1C in HD and uremic patients, it would be a reliable marker of impaired glucose tolerance.

## Conclusions

There was increased HgA1C and impaired glucose tolerance in HD and RT patients. This was accompanied by hyperlipidemia in HD patients (with hypertriglyceridemia) and RT recipients (with hypercholesterolemia). The impact upon the progression of atherosclerosis needs more study in haemodialysis and renal transplant populations at a long term follow up.

## Competing interests

None declared.

## Authors' contributions

***HA ***reviewed the literatures and wrote the manuscript and also helped to do statistical analysis, ***AN ***performed GTT and the other biochemical markers, ***MN ***participated as coordinator between laboratory and clinic, ***HTK ***selected the patients and collected data

## Pre-publication history

The pre-publication history for this paper can be accessed here:



## References

[B1] Joy MS, Cefalu WT, Hogan SL, Nachman PH (2002). Long-term glycemic control measurements in diabetic patients receiving hemodialysis. Am J Kidney Dis.

[B2] Mak RH, DeFronzo RA (1992). Glucose and insulin metabolism in uremia. Nephron.

[B3] Androgue HJ (1992). Glucose hemostasis and the kidney. Kidney Int.

[B4] Alverstrand A (1997). Carbohydrate and insulin metabolism in renal failure. Kidney Int.

[B5] Schmitz O (1985). Insulin mediated glucose uptake in nondialysed and dialysed uremic insulin dependent diabetic subjects. Diabetes.

[B6] Graf H, Prager R, Koverik J, Luger A, Schernthaner G, Pingerra WF (1985). Glucose metabolism and insulin sensitivity in patients on chronic hemodialysis. Metabolism.

[B7] Oshida Y, Sato Y, Shiraishi S, Sakamoto N (1987). Studies on glucose tolerance in chronic renal failure: estimation of insulin sensitivity before and after initiation of hemodialysis. Clin Nephrol.

[B8] Gunnarson R, Arner P, Lundgren G (1977). Steroid diabetes after transplantation: a preliminary report. Scand J Urol Nephrol.

[B9] Rao M, Jacob CK, Shastry JC (1992). Post renal transplant diabetes mellitus-a retrospective study. Nephrol Dial Transplant.

[B10] DeFronzo RA, Smith JD (1985). Is glucose intolerance harmful for the uremic patient?. Kidney Int.

[B11] Paisey R, Banks R, Holton R, Young K, Hopton M, White D, Hartog M (1986). Glycosylated haemoglobin in uraemia. Diabet Med.

[B12] Cosio FG, Pesavento TE, Osei K, Henry ML, Ferguson RM (2001). Post transplant diabetes mellitus: increasing incidence in renal allograft recipients transplanted in recent years. Kidney Int.

[B13] Burtis CA, Ashwood RA (1999). in Textbook of clinical chimestry.

[B14] American Diabetes Association (1998). Clinical Practice recommendations. Diabetes Care.

[B15] Mak RH (2000). Impact of end-stage renal disease and dialysis on glycemic control. Semin Dial.

[B16] Mak RH (1996). Insulin resistance in uremia. Pediatr Res.

[B17] Mak RH (1996). The effect of erythropoietin on insulin, amino acid and lipid metabolism in uremia. J Pediatr.

[B18] Church JM, Hill GL (1988). Impaired glucose metabolism in surgical patients improved by intravenous nutrition: assessment by euglycemic-hyperinsulinemic clamp. Metabolism.

[B19] Mak RH (1992). 1,25 dihydroxycholecalciferol corrects glucose intolerance in hemodialysis patients. Kidney Int.

[B20] Vesco L, Busson M, Bedrossian J, Bitker MO, Heisse C, Lang P (1996). Diabetes mellitus after renal transplantation: Characteristics, outcome, and risk factors. Transplantation.

[B21] Hathaway DK, Tolley EA, Blakely ML, Winsett RP, Gaber AO (1994). Development of an index to predict post-transplant diabetes mellitus. Clin Transplant.

[B22] Sumrani NB, Delaney V, Ding Z, Davis R, Daskalakis P, Friedman EA, Butt KM (1991). Diabetes mellitus after renal transplantation in the cyclosporine era – An analysis of risk factors. Transplantation.

[B23] Boudreaux JP, Mc Hugh L, Canafax DM (1987). The impact of cyclosporine and combination immunosuppression on the impact of posttransplant diabetes in renal allograft recipients. Transplantation.

[B24] Mayer AD, Dmitrewski J, Squifflet J-P, Besse T, Grabensee B, Klein B, Eigler FW, Heemann U, Pichlmayr R, Behrend M, Vanrentergham Y, Donck J, van Hooff J, Christiaans M, Morales JM, Andres A, Johnson RW, Short C, Buchholz B, Rehmert N, Land W, Schleibner S, Forsythe JL, Talbot D, Neumayer HH, Hauser I, Ericzon BG, Brattstrom C, Claesson K, Muhlbacher F, Pohanka E (1997). Multi-center randomized trial comparing tacrolimus (FK506) and cyclosporine in the prevention of renal allograft rejection: A report of the European Tacrolimus Multicenter Renal Study Group. Transplantation.

[B25] Vincenti F, Laskow DA, Neylan JF, Mendez R, Matas AJ (1996). One-year follow-up of an open-label trial of FK506 for primary kidney transplantation: A report of the US Multicenter FK506 Kidney Transplant Group. Transplantation.

[B26] (1996). Dysglycaemia and risk of cardiovascular disease. Lancet.

[B27] Maes BD, Kuypers D, Messiaen T, Evenepoel P, Mathieu C, Coosemans W, Pirenne J, Vanrenterghem YF (2001). Posttransplantation Diabetes Mellitus in FK-506-Treated Renal Transplant Recipients: Analysis of Incidence and Risk Factors. Transplantation.

[B28] Alberti KG, Zimmet PZ (1998). Definition, diagnosis and classification of diabetes mellitus and its complications. Part 1. Diagnosis and classification of diabetes mellitus. Provisional report of a WHO consultation. Diabetic Med.

